# How good is our diagnostic intuition? Clinician prediction of bacteremia in critically ill children

**DOI:** 10.1186/s12911-020-01165-3

**Published:** 2020-07-02

**Authors:** Katherine E. M. Hoops, James C. Fackler, Anne King, Elizabeth Colantuoni, Aaron M. Milstone, Charlotte Woods-Hill

**Affiliations:** 1 Department of Anesthesiology and Critical Care Medicine, Johns Hopkins University School of Medicine, Baltimore, MD USA; 2 Department of Pediatrics, Johns Hopkins University School of Medicine, Baltimore, MD USA; 3 Department of Biostatistics, Johns Hopkins Bloomberg School of Public Health, Baltimore, MD USA; 4 Department of Anesthesiology and Critical Care Medicine, Children’s Hospital of Philadelphia, Philadelphia, PA USA

**Keywords:** Sepsis, Bacteremia, Quality improvement, Stewardship, Decision support, Prediction

## Abstract

**Background:**

Clinical intuition and nonanalytic reasoning play a major role in clinical hypothesis generation; however, clinicians’ intuition about whether a critically ill child is bacteremic has not been explored. We endeavored to assess pediatric critical care clinicians’ ability to predict bacteremia and to evaluate what affected the accuracy of those predictions.

**Methods:**

We conducted a retrospective review of clinicians’ responses to a sepsis screening tool (“Early Sepsis Detection Tool” or “ESDT”) over 6 months. The ESDT was completed during the initial evaluation of a possible sepsis episode. If a culture was ordered, they were asked to predict if the culture would be positive or negative. Culture results were compared to predictions for each episode as well as vital signs and laboratory data from the preceding 24 h.

**Results:**

From January to July 2017, 266 ESDTs were completed. Of the 135 blood culture episodes, 15% of cultures were positive. Clinicians correctly predicted patients with bacteremia in 82% of cases, but the positive predictive value was just 28% as there was a tendency to overestimate the presence of bacteremia. The negative predictive value was 96%. The presence of bandemia, thrombocytopenia, and abnormal CRP were associated with increased likelihood of correct positive prediction.

**Conclusions:**

Clinicians are accurate in predicting critically ill children whose blood cultures, obtained for symptoms of sepsis, will be negative. Clinicians frequently overestimate the presence of bacteremia. The combination of evidence-based practice guidelines and bedside judgment should be leveraged to optimize diagnosis of bacteremia.

## Background

Machine learning and artificial intelligence are now commonly applied to large datasets to predict myriad conditions [1]. These mathematical techniques promise to identify data patterns correlating to a particular physiologic syndrome or disease earlier than can even expert clinicians. We have previously shown that senior clinicians see more complete patterns than do junior clinicians [2]. Some have suggested in some circumstances the math is better than clinicians [3].

Expert intuition fundamentally relies on patterns that humans see that cannot (yet) be fully deconstructed, parameterized, computed and supported [4, 5]. Whether heuristics or intuition is more important depends on the situation; rarely should either be considered a better approach. Given the problem of human (even experts) biases, reliance on math and heuristics is appealing.

Sepsis due to bacterial bloodstream infections in children offers a particularly interesting domain in which to explore the role of clinical intuition in detecting presence of disease. This condition offers a unique challenge to clinicians: its significant prevalence, morbidity, and mortality demand rapid and accurate diagnosis, but its non-specific signs and symptoms, and its overlap with many non-infectious conditions, present real limitations to our diagnostic accuracy [6, 7]. A wealth of machine learning and electronic medical record-based algorithms have attempted to optimize early recognition of sepsis, yet clinicians’ intuition about whether a critically ill child is bacteremic has not been well explored [8–10]. The literature supports that clinical intuition and nonanalytic reasoning play a major role in hypothesis generation for providers of all experience levels, from novices in familiar scenarios to experts identifying patterns and creating differential diagnoses [2, 11, 12]. One recent study demonstrated that certain features of wellness (such as age appropriate verbalization and playfulness) were highly reassuring to a majority of experienced clinicians evaluating children for sepsis [13]. We do not believe that predictive analytics can, or should [14], fully supersede the contributions of a clinician in making an accurate diagnosis, but data exploring this is quite limited.

A cornerstone of clinical evaluation for suspected bacterial sepsis is a blood culture. Ordering a blood culture is, fundamentally, a hypothesis-driven action: a culture is ordered when a clinician believes their patient may have a bacterial bloodstream infection, and testing is indicated to confirm or exclude an infection. Unfortunately, little data and few guidelines exist to aid clinicians in determining appropriate versus inappropriate indications for ordering blood cultures, despite blood cultures being used frequently in pediatric intensive care units (PICU), emergency departments, and clinics [15–18]. Blood cultures have an overall yield of only a 5–15% and up to a 50% false positive rate [7–9]. Blood cultures are clearly indicated for patients with signs or symptoms of sepsis, but indiscriminate use of blood cultures can lead to avoidable false positive results, unnecessary antibiotics, increased lengths of stay, and increased costs [10].

We implemented a quality improvement program to improve early sepsis detection and reduce unnecessary blood culture use in our pediatric intensive care unit. In this program, we assessed pediatric critical care clinicians’ a priori ability to correctly predict blood culture results for children being evaluated for sepsis; i.e. examining clinician intuition specifically related to the presence of bloodstream infection. We then sought to identify factors associated with the clinician’s prediction of whether a blood culture was positive or negative. Our ultimate goal is to augment sepsis prediction algorithms and data-driven decision support tools with clinical intuition, an innovative approach that may both facilitate continued appropriate use of diagnostic tools like blood cultures, while encouraging important efforts to minimize unnecessary testing. The first step in this approach is, therefore, to investigate this clinical intuition and determine how accurate PICU clinicians may be in predicting presence or absence of bacteremia.

## Methods

Since 2014, the 36-bed multidisciplinary PICU in our academic tertiary care center has implemented a quality improvement program to improve early recognition of sepsis while also reducing the unnecessary use of blood cultures [19]. In the context of judicious blood culture use, a novel sepsis screening tool (“Early Sepsis Detection Tool” or “ESDT”) was created to provide decision support to clinicians about symptoms, risk factors, and evaluation for bacterial sepsis (Supplement A). The ESDT enumerated signs of sepsis (e.g., fever, rigors, hypotension, poor perfusion, metabolic acidosis, leukocytosis) and risk factors for sepsis (e.g., immune compromise, presence of a central venous catheter) as well as signs of focal infections (e.g., isolated respiratory symptoms, dysuria, wound erythema) and noninfectious sources of fever (e.g., surgery within 24 h, narcotic withdrawal). It also included definitions of sepsis and septic shock. This project was acknowledged by the Johns Hopkins Medicine Institutional Review Board as quality improvement.

For a six-month period, from January through July of 2017, an ESDT was completed by clinicians during the initial phase of evaluation of a possible sepsis episode. Clinicians were prompted to complete an ESDT when a patient was febrile, hypothermic, or had evidence of poor perfusion. These prompts came from usual unit clinical workflow (i.e., bedside nurses or routine electronic medical record flowsheet data made clinicians aware of concerning symptoms or changes in patient status, such as fever, hypothermia, or change in perfusion). No automated single parameter alarm or multi-parameter alerts were in place; however, a daily assessment of the incidence of fever, hypothermia, and blood culture orders among PICU patients was conducted, and the clinicians caring for those patients were also contacted directly by project personnel (KH) to ensure compliance completing the ESDT. The child’s clinicians including the bedside nurse, resident or nurse practitioner, fellow, and/or attending together indicated on the ESDT form what risk factors for sepsis were present and whether a blood culture was ordered. The clinical team was of variable composition depending on time of day and individual availability; no effort was made to alter the routine workflows. In our institution, attending physicians and fellows rotate weekly, and frontline providers and nurses often rotate daily or every few days in their care for a given patient. No identifying information was collected from the clinicians, so it is unknown precisely how many unique clinicians are represented in this sample. If a culture was ordered, the clinicians (bedside nurse, frontline provider, fellow, and attending) were each asked to individually predict if the culture would be positive or negative at the time that the order was placed and write the predictions on a single ESDT form.

We compared each provider’s prediction to the “gold standard” (the blood culture result) and calculated sensitivity, specificity, positive predictive value, and negative predictive value and corresponding 95% confidence intervals for each clinician type and for clinicians in aggregate. Fisher’s exact tests were used to assess for significant differences among the clinician groups with respect to sensitivity, specificity, positive predictive value, and negative predictive value.

**Fig. 1:**
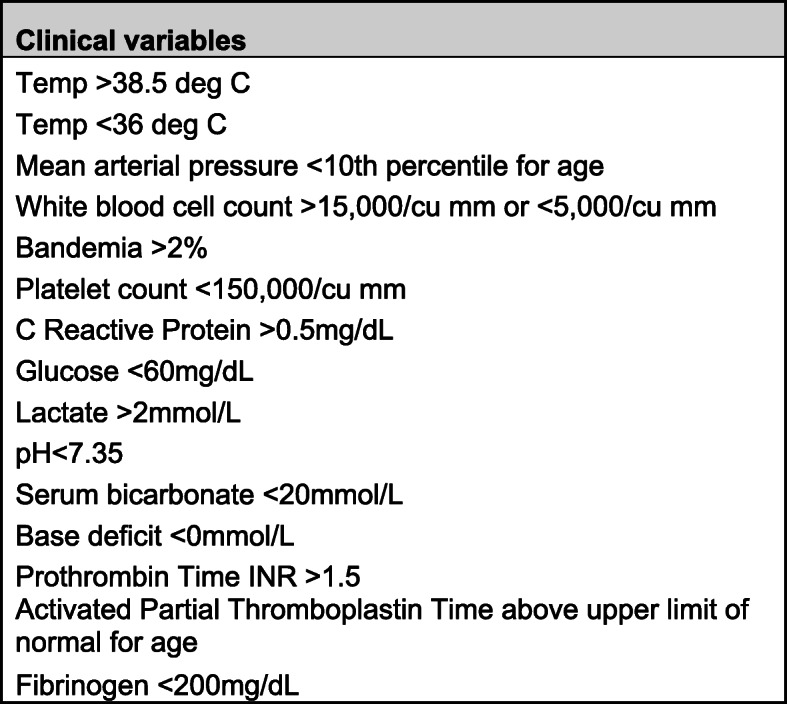
Clinical Variables. Clinical data collected from the 24 h prior to a blood culture being obtained and the threshold beyond which a value was called abnormal in subsequent analyses. Abbreviations: Temp temperature; INR International normalized ratio

We constructed logistic regression models to describe the association between clinical variables including blood pressure, lactemia, and bandemia (see Fig. 1 for a complete list of the clinical variables) and a clinician’s prediction of culture results. We then separately constructed additional models to evaluate the association between clinical variables and the accuracy or correctness of those clinical predictions. Predictions were considered correct if a clinician predicted that a given culture would be positive and that blood culture did grow bacteria or yeast or if a clinician predicted that a blood culture would be negative and that blood culture had no growth on its final report. All providers were assumed to have been aware of these data at the time blood cultures were ordered both because the clinical variables we examined were routinely verbalized on rounds in our unit as part of a rounding script and because a chart review is routinely performed when cultures are ordered. Continuous variables were transformed to binary code (normal or abnormal) to reflect clinically relevant thresholds such as standard definitions for fever or hypothermia or in accordance with sepsis guidelines. We used robust standard error calculations to account for multiple observations per patient and lack of independence among the observations. Because only a minority of patients (in the case of fibrinogen, only 10) had coagulation studies drawn in the 24 h before the ESDT was completed, these variables were excluded. We were unable to evaluate hypoglycemia as an exposure as none of the patients with hypoglycemia were predicted to have positive blood cultures. Data were analyzed using Stata 14.2 and VassarStats.net.

## Results

Between January and July of 2017, we collected ESDTs from 266 possible sepsis episodes, roughly half of which included a blood culture order (135/266). There were 131 cases in which an ESDT was completed but a culture was not ordered. Not all provider types completed the ESDT form for each of the 266 possible sepsis episodes, so for the 135 ESDTs with blood cultures collected, a total of 398 predictions were recorded (84 nursing encounters, 85 front line provider encounters, 141 fellow encounters, and 88 attending encounters), as detailed in Table 1. Of the 135 blood cultures sent at the time of ESDT completion, 20 were positive (15%). None were believed to be contaminants.

**Table 1:** Clinician predictions

Provider Predictions		Blood Culture Result		Sensitivity (95% CI)	Specificity (95% CI)	Positive Predictive Value (95% CI)	Negative Predictive Value (95% CI)
		Positive (n)	Negative (n)				
Overall Prediction of Culture Result	Positive (n)	41	107	82% (0.68,0.91)	69% (0.64, 0.74)	28% (0.21, 0.36)	96% (0.93, 0.98)
	Negative (n)	9	241				
RN Prediction of Culture Result	Positive (n)	9	29	90% (0.54,0.99)	61% (0.49, 0.72)	24% (0.12, 0.41)	98% (0.87, 0.99)
	Negative (n)	1	45				
FLP Prediction of Culture Result	Positive (n)	6	26	86% (0.42, 0.99)	67% (0.55, 0.77)	19% (0.08, 0.37)	98% (0.89, 0.99)
	Negative (n)	1	52				
Fellow Prediction of Culture Result	Positive (n)	17	30	81% (0.57, 0.94)	75% (0.66, 0.82)	36% (0.23, 0.52)	96% (0.89, 0.99)
	Negative (n)	4	90				
Attending Prediction of Culture Result	Positive (n)	9	22	75% (0.43, 0.93)	71% (0.59, 0.81)	29% (0.15, 0.48)	95% (0.84, 0.99)
	Negative (n)	3	54				

Blood culture results compared to clinician prediction and characteristics of clinician prediction, separated by provider type. This table illustrates a series of classic “2 × 2” tables to calculate test characteristics sensitivity, specificity, positive and negative predictive values. For example, among RNs, the sensitivity was 90% or 9 of 10 positive cultures were correctly predicted to be positive. Abbreviations: RN *registered nurse,* FLP front line provider (in our unit, a *resident or nurse practitioner),* n *number of observations,* CI confidence interval

Of the 398 predictions, clinicians’ predictions were correct 71% of the time (282/398). Overall, clinicians demonstrated a 69% specificity (i.e. clinicians predicted a negative blood culture when the blood culture was ultimately negative 241/348 times). The negative predictive value (i.e. the proportion of negative predictions which are in fact negative cultures) was high at 96% (241/250 negative predictions). Clinicians correctly predicted bacteremia in 82% of positive blood cultures (41/50 cultures) (i.e. sensitivity), however, given the relatively low prevalence of positive cultures, the probability of a blood culture being positive for bacteria if predicted to be positive was 28% (41/148 positive predictions) (i.e. positive predictive value) (Table 1). Out of 266 possible sepsis episodes, there were 6 patients for whom one or more clinicians predicted a negative culture, but that patient in fact subsequently had bacteremia. Alternatively, there were 107 times (27% of all predictions) in which a clinician predicted a positive culture in a patient who did not have bacteremia. When comparing the predictions of individual clinician types, no difference reached statistical significance.

Next, we examined whether available clinical and laboratory data at the time of blood culture collection was associated with clinician predictions. These data were analyzed to examine the relationship between the clinical variables present in the 24 h preceding blood culture orders, and the provider predictions for each culture, in order to determine which variables were associated with higher likelihood of a positive prediction. Based on simple logistic regression comparing provider prediction to the presence or absence of a certain clinical variable, the presence of bandemia (Odds Ratio OR: 4.44, *p* < 0.001), an abnormal CRP (OR: 8.73, *p* = 0.001), and lactemia (OR: 4.19, *p* = 0.011) were associated with a higher likelihood of predicting that a culture would be positive (Table 2).

**Table 2:** Comparison of clinical variables to predictions

Clinical variables	Predict negative blood culture (n)	Predict positive blood culture (n)	OR (95% CI)	*p*-value
Afebrile	120	78		
Febrile	130 (52%)	70 (47%)	0.83 (0.356, 1.927)	*p* = 0.662
Normothermia	193	126		
Hypothermia	57 (22%)	18 (13%)	0.48 (0.228, 1.027)	*p* = 0.059
Normotension	175	83		
Hypotension	67 28%	40 (33%)	1.26 (0.477, 3.322)	*p* = 0.642
Normal WBC count	92	63		
Abnormal WBC count	90 (49%)	68 (52%)	1.10 (0.419, 2.904)	*p* = 0.842
No bandemia	81	23		
Bandemia	46 (36%)	58 (72%)	4.44 (1.970, 10.009)	***p*** **< 0.001**
Normal Plt count	111	62		
Thrombocytopenia	71 (39%)	69 (53%)	1.74 (0.792, 3.821)	*p* = 0.168
Normal CRP	29	3		
Abnormal CRP	72 (71%)	65 (96%)	8.73 (2.581, 29.501)	***p*** **= 0.001**
Normal lactate	55	25		
Lactemia	21 (28%)	40 (62%)	4.19 (1.383, 12.699)	***p*** **= 0.011**
Normal pH	56	43		
Acidosis	83 (60%)	59 (58%)	0.93 (0.421, 2.037)	*p* = 0.848
Normal HCO3	116	78		
Low HCO3	23 (17%)	24 (24%)	1.55 (0.468, 5.141)	*p* = 0.472
No base deficit	82	51		
Base deficit	61 (43%)	50 (50%)	1.32 (0.519, 3.349)	*p* = 0.562

Results of unadjusted logistic regression analysis comparing clinical variables to provider predictions of culture results. Abbreviations: WBC *white blood cell count,* Plt *platelet,* CRP C-reactive pro*tein,* HCO3 *serum bicarbonate,* CI confidence interval

On further analysis, we constructed a logistic regression model for the accuracy of providers’ predictions in order to determine which variables were associated with higher likelihood of a correct prediction (either correctly predicted to be positive or correctly predicted to be negative).

Providers were more likely to correctly predict that a culture will be positive in the presence of thrombocytopenia (OR 21.6, 95% CI 1.88 to 247.7, *p* = 0.014). Because there were neither incorrect negative predictions among patients with bandemia or lactemia nor correct positive predictions among patients without bandemia or abnormal CRP, we were unable to assess an odds ratio for each subgroup; however, as an aggregate, the presence of bandemia and abnormal CRP were associated with more accurate prediction of positive cultures (*p* = 0.003 for bandemia, *p* = 0.018 for abnormal CRP overall) (Supplement B).

Subsequently, we further analyzed the negative predictions to assess for variables affecting the accuracy of negative prediction that might have been lost in the aggregate analysis (shown in Table 3). Some of the variables were omitted from the analysis due to an absence of incorrect negative predictions (predicting a negative culture in a patient who was bacteremic). None of the variables were associated with the accuracy of negative prediction.

**Table 3:** Comparison of clinical variables to correctness of predictions

Clinical variables	Correctly predict negative blood culture n (%)	Incorrectly predict negative blood culture n (%)	OR (95% CI)	*p*-value
Afebrile	114	6		
Febrile	127 (61%)	3 (33%)	2.23 (0.60, 8.22)	0.229
Normothermia	189	4		
Hypothermia	52 (22%)	5 (56%)	0.22 (0.03, 1.64)	0.139
Normotension	170	5		
Hypotension	63 (27%)	4 (44%)	0.46 (0.04, 5.17)	0.532
Normal WBC count	88	4		
Abnormal WBC count	85 (49%)	5 (56%)	0.77 (0.09, 6.33)	0.81
No bandemia	81	0		
Bandemia	46 (36%)	0		
Normal Plt count	103	8		
Thrombocytopenia	70 (40%)	1 (11%)	5.44 (0.50, 58.88)	0.164
Normal CRP	28	1		
Abnormal CRP	66 (70%)	6 (86%)	0.39 (0.03, 5.83)	0.497
Normal lactate	51	4		
Lactemia	21 (29%)	0 (0%)		
Normal pH	49	7		
Acidosis	81 (62%)	2 (22%)	5.79 (0.72, 46.63)	0.099
Normal HCO3	107	9		
Low HCO3	23 (18%)	0 (0%)		
No base deficit	74	8		
Base deficit	60 (45%)	1 (11%)	6.49 (0.60, 70.65)	0.125

Results of logistic regression analysis comparing clinical variables to correctness of negative provider predictions of culture results. *Abbreviations*: *WBC* white blood cell count, *Plt* platelet, *CRP* C-reactive protein, *HCO3* serum bicarbonate, *n* number of observations, *CI* confidence interval

## Discussion

Despite a growing body of evidence demonstrating the importance of early detection and treatment of bacterial sepsis, comparatively little work has been done to understand what influences clinician decision making related to evaluation for bacteremia. Although blood cultures are perceived as low-risk tests, excessive diagnostic testing is increasingly recognized as a component of medical overuse that can harm patients [20–22]. Prioritizing the de-adoption of unnecessary clinical practices is now recognized by professional societies and governments as an integral component to the delivery of high-value care [23–26]. For blood cultures in particular, patient harm may result from false positive results, unnecessary antibiotics, increased lengths of stay, and increased costs [17]. In recognition of this fact, a better understanding of the complex decision-making process clinicians employ in their diagnostic approach to possible bacteremia is urgently needed.

Our data reveals that pediatric critical care clinician predictions of blood culture results are accurate with a strong sensitivity (> 80%), a robust negative predictive value (96%), and a failure to predict bacteremia in only 4% of cases. This investigation represents the first attempt to quantify the predictive abilities of pediatric critical care clinicians specifically focusing on the presence or absence of bacteremia, and it offers several important insights that warrant further exploration. Given prior work demonstrating that clinicians require a sensitivity of nearly 100% to accept sepsis prediction rules [27], intuition and clinical judgement should be insufficient to predict bacteremia. This highlights the continued need for rigorous study of prediction models and how they may best be incorporated into clinical decision-making.

First, approximately 15% of cultures in our study yielded positive results, which aligns with previous literature demonstrating a 5–15% positivity rate of blood cultures [6, 7]. When viewed together with the high negative predictive value of clinician pre-test estimation of culture results, an important question emerges: why do clinicians order a test with generally negative results when they appear to be highly skilled in predicting this negative result? It is almost certain that a mental model of bacterial sepsis as a “cannot miss” diagnosis drives this behavior, and the mortality and morbidity from delayed sepsis therapy appears to be the rationale for this approach. Clinicians often predict a patient is bacteremic who ultimately has a negative blood culture. In our sample, of the 348 negative blood cultures, 107 were predicted to have been positive. It is increasingly accepted that rapid diagnosis and treatment of sepsis affects outcomes, and clinical practices have shifted accordingly [28]. However, the unintended negative consequences of unnecessary testing for bacteremia (particularly the false-positive results and the antibiotic initiation that typically is paired with this testing) must also be a part of our clinical decision-making process. Exploring what drives clinicians to accurately predict the absence of bacteremia may reveal novel and important strategies to further reduce unnecessary blood cultures and minimize some of these unintended consequences.

Second, the presence of certain clinical factors was associated with prediction category (positive or negative) and prediction accuracy. While limited by sample size and the overall low prevalence of bacteremia, the presence of bandemia, thrombocytopenia, and abnormal CRP were associated with increased likelihood of correct positive prediction. Prior attempts to determine whether inflammatory markers such as C-reactive protein or procalcitonin, white blood cell count values or percent of immature neutrophils can reliably distinguish bacterial infection in a pediatric ICU population, have found mixed results [29]. There are currently no widely accepted guidelines for how or when to use these biomarkers or laboratory values in critically ill children. While our limited data certainly do not support any specific conclusions about the role of these laboratory tests in indicating the presence of bacteremia, further exploration of how clinicians utilize these ancillary laboratory tests when considering diagnostic evaluation for bacteremia should be performed, with an eye to developing consensus guidelines that could better standardize their use.

Prediction of sepsis, death from sepsis, and the utility of blood cultures has been investigated for decades. Again, the application of novel machine learning algorithms raises the possibility of finding patterns in the data before even expert clinicians can make a diagnosis of sepsis. Rather, our intent was to understand what data clinicians most use so that solutions joining clinical intuition with machine learning algorithms can most effectively complement the other’s strengths.

Finally, diagnostic stewardship, defined as refining the use of diagnostic tools in order to improve treatment decisions, is an essential component of our healthcare system’s transformation to a high value care model [30]. The ability of diagnostic stewardship to alter downstream treatment decisions around antibiotics is beginning to emerge [19, 31]. Study of exactly what drives clinicians to obtain blood cultures in the pediatric ICU may demonstrate specific examples of low-risk scenarios wherein cultures can safely be avoided and inform creation of generalizable, evidence-based criteria to standardize how this test is used in critically ill children, on a large scale. Leveraging these two insights to refine decision making around blood cultures may not only optimize diagnosis of bacteremia in the PICU, ensuring that at-risk children are appropriately tested, but enhance antibiotic stewardship efforts as well. This connection warrants further investigation.

Our study must be interpreted in the context of several limitations. Because no automated single parameter alarm or multi-parameter alerts were in place, it is unknown how many opportunities to complete an ESDT were missed. Further, predictive ability is influenced by prevalence of a disease in a population. Here our intent was to understand the factors clinicians use to confirm an alert. In settings where the prevalence of sepsis are lower (e.g. acute care inpatient or emergency department) clinicians likely have different risk thresholds. In our PICU setting, with a program already in place to reduce unnecessary blood cultures, the high threshold for ordering blood cultures may have affected the predictive ability of clinicians and may limit this study’s generalizability. In a setting where the threshold for obtaining blood cultures is lower, the negative predictive ability may be similar, but the positive predictive ability may be even lower. We were not able to collect predictions from all types of providers in equal proportions to allow detection of statistically significant differences in prediction ability across provider type, nor did we attempt to blind the different clinicians participating in the assessment to each other’s predictions, which limits our ability to differentiate among the predictive abilities of various clinical disciplines. The absolute number of positive blood cultures was low, limiting our ability to analyze associations between pre-culture clinical data and clinician’s predictive accuracy. Finally, it is unknown to what degree predictions by individuals or groups of clinicians may have changed over time during the study period; no feedback on clinicians’ performance was provided by the study team, but clinicians were likely often aware of the concordance of their predictions with the blood culture result.

## Conclusions

Pediatric critical care clinicians’ a priori predictions of blood culture results have a high negative predictive value in predicting the absence of bacteremia. This finding combined with the low likelihood of blood culture positivity in this population suggest that continued refinement of decision support tools focused on early sepsis recognition and diagnosis could transform the use of blood cultures. Improved decision making about blood culture ordering may reduce empiric broad-spectrum antibiotics use in the PICU. Linking diagnostic stewardship to treatment stewardship in this manner has as-yet untapped power as a high value care initiative.

## Supplementary information

**Additional file 1: Supplement A.** Early Sepsis Detection Tool, a cognitive aid for the diagnosis of sepsis in critically ill children.

**Additional file 2: Supplement B.** Results of logistic regression analysis comparing clinical variables to correctness of provider predictions of culture results.

## Data Availability

The datasets used and/or analyzed during the current study are available from the corresponding author on reasonable request.

## Abbreviations

PICU: Pediatric Intensive Care Unit
ICU: Intensive Care Unit
ESDT: Early Sepsis Detection Tool
KH: Katherine Hoops
RN: Registered Nurse
FLP: Front Line Provider
WBC: White blood cell count
Plt: Platelet
CRP: C-reactive protein
HCO3: Bicarbonate
n: number of observations
CI: Confidence interval
