# Supplementary material for: How good is our diagnostic intuition? Clinician prediction of bacteremia in critically ill children
Source: BMC Med Inform Decis Mak. 2020 Jul 2;20:144. doi: 10.1186/s12911-020-01165-3 (PMC7330962; doi:10.1186/s12911-020-01165-3)
Supplement: Supplementary file 1 — Additional file 1: Supplement A. Early Sepsis Detection Tool, a cognitive aid for the diagnosis of sepsis in critically ill children. [file 12911_2020_1165_MOESM1_ESM.docx]

**Early Sepsis Detection Tool**

Instructions: Please complete this form when evaluating a patient for possible bacteremia or sepsis. Bedside RN and frontline provider complete this together, ideally at bedside.

**Circle One:**

NP RN Resident (Year: 2, 3) Hospitalist Fellow (Year: 1, 2, 3) Attending

**If sepsis is not suspected:**

1. Consider other sources of infection on exam/history:
   1. Conjunctivitis
   2. Otitis media
   3. Pharyngitis
   4. Respiratory symptoms
   5. Increased trach or ETT secretions
   6. Urine color/consistency change/dysuria
   7. Diarrhea (>3 stools/24 hours)
   8. Superficial wound erythema/drainage/cellulitis without any of symptoms in item 1
2. Patient has non-infectious cause of symptoms
   1. Withdrawal – recent sedation weans? Elevated WAT score?
   2. Feeding intolerance causing tachycardia, emesis, diarrhea
   3. Surgery within last 24 hours
3. Negative blood cultures drawn within last 24-48 hours, and no clinical change in the patient other than fever

**Sepsis may be suspected if:**

1. Signs of systemic infection
   1. Temperature: max min source? (*Rectal temp is contraindicated in neutropenic pt.)
   2. Rigors
   3. Unexplained tachycardia
   4. Hypotension
   5. Poor perfusion
   6. Metabolic acidosis
   7. Elevated WBC from baseline
   8. Elevated or uptrending CRP
   9. Already on antibiotics but persistent fever or clinical symptoms?
2. Risk Factors
   1. Host Immune Status: immune compromised
   2. Central Line present AND concern for:
      1. Symptoms (e.g. hypotension) when infusing through the line
      2. Line site inflamed, tender, purulent?
      3. Consider duration of line – abx-coated PICC>56 days or abx-coated Cook >28 days?
      4. Concern for line contamination?

**Patient Sticker**

Date Pt. Assessed: __________________

**Sepsis Criteria:**

**Sepsis:** A life threatening organ dysfunction caused by a dysregulated host response to infection.

**Septic Shock**: Hypotension requiring vasopressors and having lactate level ≥ 2 despite volume resuscitation

After completion of this tool, is a blood culture ordered?

**Circle One:**

**YES** **NO**

**(ALSO COMPLETE BACK SIDE OF THIS FORM)**

**FLP**

Please complete ESD, circling applicable clinical signs or risk factors.

**RN**

What was the trigger to notify a provider?

Circle all that apply:

Fever Hypothermia

Hypotension Poor Perfusion

| ***PLEASE COMPLETE:*** | Agree | Disagree |
| --- | --- | --- |
| I believe pt. has bacteremia | 1 | 2 |
| I believe pt. will have progressive physiologic derangement due to sepsis within the next 48 hours. | 1 | 2 |
| I believe this pt. is in shock | 1 | 2 |

| ***PLEASE COMPLETE:*** | Agree | Disagree |
| --- | --- | --- |
| I believe pt. has bacteremia | 1 | 2 |
| I believe pt. will have progressive physiologic derangement due to sepsis within the next 48 hours. | 1 | 2 |
| I believe this pt. is in shock | 1 | 2 |

**Clinician Signature:** __________________________

**Clinician Signature:** ____________________________

| ***PLEASE COMPLETE:*** | Agree | Disagree |
| --- | --- | --- |
| I believe pt. has bacteremia | 1 | 2 |
| I believe pt. will have progressive physiologic derangement due to sepsis within the next 48 hours. | 1 | 2 |
| I believe this pt. is in shock | 1 | 2 |

| ***PLEASE COMPLETE:*** | Agree | Disagree |
| --- | --- | --- |
| I believe pt. has bacteremia | 1 | 2 |
| I believe pt. will have progressive physiologic derangement due to sepsis within the next 48 hours. | 1 | 2 |
| I believe this pt. is in shock | 1 | 2 |

**Fellow**

Clinical Synopsis:

________________________________________________________________________________________________________________________________________________________________________________________

**Attending**

Additional Notes:

________________________________________________________________________________________________________________________________________________________________________________________

****Place completed EDST in manilla envelope in PICU team rooms**
