# Supplementary material for: How good is our diagnostic intuition? Clinician prediction of bacteremia in critically ill children
Source: BMC Med Inform Decis Mak. 2020 Jul 2;20:144. doi: 10.1186/s12911-020-01165-3 (PMC7330962; doi:10.1186/s12911-020-01165-3)
Supplement: Supplementary file 2 — Additional file 2: Supplement B. Results of logistic regression analysis comparing clinical variables to correctness of provider predictions of culture results. [file 12911_2020_1165_MOESM2_ESM.docx]

| Predictions | Clinical variables | | OR (95% CI) | p-value |
| --- | --- | --- | --- | --- |
|  | Afebrile n(%) | Febrile n(%) |  | 0.369 |
| Incorrect Positive Prediction | 52 (13.1%) | 55 (13.8%) | 2.12 (0.54, 8.22) | 0.279 |
| Incorect Negative Prediction | 6 (1.5%) | 3 (0.8%) |  |  |
| Correct Positive Prediction | 26 (6.5%) | 15 (3.8%) | 1.15 (0.27, 4.91) | 0.846 |
| Correct Negative Prediction | 114 (28.6%) | 127 (31.9%) | 2.23 (0.61, 8.22) | 0.229 |
|  | Normothermia n(%) | Hypothermia n(%) |  | 0.0588 |
| Incorrect Positive Prediction | 91 (23%) | 12 (3%) | 0.11 (0.01, 1.08) | **0.03** |
| Incorect Negative Prediction | 4(1%) | 5 (1.3%) |  |  |
| Correct Positive Prediction | 35 (8.9%) | 6 (1.5%) | 0.14 (0.02, 0.82) | 0.936 |
| Correct Negative Prediction | 189 (48%) | 52 (13.2%) | 0.22 (0.03, 1.64) | 0.139 |
|  | Normotension n(%) | Hypotension n(%) |  | 0.91 |
| Incorrect Positive Prediction | 58 (14.2%) | 24 (6.6%) | 0.52 (0.04, 7.4) | 0.627 |
| Incorect Negative Prediction | 5 (1.4%) | 4 (1.1%) |  |  |
| Correct Positive Prediction | 25 (6.8%) | 16 (4.4%) | 0.80 (0.06, 10.32) | 0.864 |
| Correct Negative Prediction | 170 (46.6%) | 63 (17.3%) | 0.46 (0.04, 5.17) | 0.532 |
|  | Normal WBC count n(%) | Abnl WBC count n(%) |  | **0.031** |
| Incorrect Positive Prediction | 32 (10.2%) | 62 (19.8%) | 1.55 (0.22, 10.91) | 0.66 |
| Incorect Negative Prediction | 4 (1.3%) | 5 (1.6%) |  |  |
| Correct Positive Prediction | 31 (9.9%) | 6 (1.9%) | 0.15 (0.02, 0.97) | **0.046** |
| Correct Negative Prediction | 88 (28.1%) | 85 (27.2%) | 0.77 (0.09,6.32) | 0.81 |
|  | No bandemia n(%) | Bandemia n(%) |  | **0.001** |
| Incorrect Positive Prediction | 23 (11.1%) | 44 (21.2%) |  |  |
| Incorect Negative Prediction | 0 (0%) | 0 (0%) |  |  |
| Correct Positive Prediction | 0 (0%) | 14 (6.7%) |  |  |
| Correct Negative Prediction | 81 (38.9%) | 46 (22.1%) | 0.30 (0.13, 0.68) | **0.004** |
|  | Normal Plt count n(%) | Thrombocytopenia n(%) |  | **0.026** |
| Incorrect Positive Prediction | 52 (16.6%) | 43 (13.4%) | 6.46 (0.76, 54.82) | 0.087 |
| Incorect Negative Prediction | 8 (2.6%) | 1 (0.3%) |  |  |
| Correct Positive Prediction | 10 (3.2%) | 27 (8.6%) | 21.60 (1.88, 247.7) | **0.014** |
| Correct Negative Prediction | 103 (32.9%) | 70 (22.4%) | 5.44 (0.50, 58.79) | 0.163 |
|  | Normal CRP n(%) | Abnormal CRP n(%) |  | **0.018** |
| Incorrect Positive Prediction | 3 (1.8%) | 45 (26.6%) | 2.50 (0.19, 33.39) | 0.488 |
| Incorect Negative Prediction | 1 (0.6%) | 6 (3.6%) |  |  |
| Correct Positive Prediction | 0 (0%) | 20 (11.8%) |  |  |
| Correct Negative Prediction | 28 (16.6%) | 66 (39%) | 0.39 (0.03, 5.8) | 0.496 |
|  | Normal lactate n(%) | Lactemia n(%) |  | **0.035** |
| Incorrect Positive Prediction | 23 (16.3%) | 25 (17.7%) |  |  |
| Incorect Negative Prediction | 4 (2.8%) | 0 (0%) |  |  |
| Correct Positive Prediction | 2 (1.4%) | 15 (10.6%) | 6.90 (0.60, 79.40) | 0.121 |
| Correct Negative Prediction | 51 (36.2%) | 21 (14.9%) | 0.38 (0.12, 1.17) | 0.091 |
|  | Normal pH n(%) | Acidosis n(%) |  | 0.422 |
| Incorrect Positive Prediction | 26 (10.8%) | 37 (15.4%) | 4.98 (0.69, 36.16) | 0.112 |
| Incorect Negative Prediction | 7 (2.9%) | 2 (0.8%) |  |  |
| Correct Positive Prediction | 17 (7.1%) | 22 (9.1%) | 4.53 (0.55, 37) | 0.159 |
| Correct Negative Prediction | 49 (20.3%) | 81 (33.6%) | 5.79 (0.72, 46.46) | 0.099 |
|  | Normal HCO3 n(%) | Low HCO3 n(%) |  | 0.291 |
| Incorrect Positive Prediction | 43 (17.8%) | 20 (8.3%) |  |  |
| Incorect Negative Prediction | 9 (3.7%) | 0 (0%) |  |  |
| Correct Positive Prediction | 35 (14.5%) | 4 (1.7%) | 0.25 (0.02, 3.10) | 0.278 |
| Correct Negative Prediction | 107 (44.4%) | 23 (9.6%) | 0.46 (0.15, 1.41) | 0.174 |
|  | No base deficit n(%) | Base deficit n(%) |  | 0.189 |
| Incorrect Positive Prediction | 28 (11.5%) | 35 (14.3%) | 10.00 (1.06, 94.44) | **0.044** |
| Incorect Negative Prediction | 8 (3.3%) | 1 (0.4%) |  |  |
| Correct Positive Prediction | 23 (9.4%) | 15 (6.2%) | 5.20 (0.34, 79.23) | 0.234 |
| Correct Negative Prediction | 74 (30.3%) | 60 (24.6%) | 6.49 (0.60, 70.38) | 0.124 |
